# Blood Product Management: A Comprehensive Analysis in a Trauma Center Hospital

**DOI:** 10.34172/aim.31324

**Published:** 2025-02-01

**Authors:** Tarannom Mohammadtaghi Kashi, Mohammad Taher Hojjati, Ali Asghar Ayatollahi

**Affiliations:** ^1^Laboratory Sciences Research Center, Golestan University of Medical Sciences, Gorgan, Iran

**Keywords:** Blood products, Blood transfusion, Cross-match, Cross-match to transfusion ratio, Probability of transfusion, Transfusion request

## Abstract

**Background:**

Today, excessive demand for blood products is a common issue across various medical departments. This demand can lead to improper distribution, waste of blood products, and poor availability for emergency patients. Additionally, it increases costs and the workload of blood banks, disrupting the proper distribution of products among medical centers. Ultimately, all these factors contribute to a decrease in the quality of blood products. This study was conducted to investigate the pattern of blood requests and utilization at 5-Azar Educational Hospital in the Golestan province in 2022.

**Methods:**

This cross-sectional study was conducted from June to August 2022. The study population comprised all patients who requested blood product reservations upon admission to the hospital.

**Results:**

In the present study, 514 patients with requests for blood product reservations were included, resulting in 739 cross-matches and ultimately 677 transfusions. The cross-match to transfusion (C/T) ratio for this study was 1.09, while the probability of transfusion (T %) was 91.6%. The highest incidence of blood reservation wastage was associated with the neurosurgery department.

**Conclusion:**

By addressing the identified challenges and promoting adherence to guidelines, healthcare institutions can improve patient outcomes and ensure responsible use of blood resources.

## Introduction

 The demand for blood and blood products is increasing, and raising concerns about the insufficient availability of these resources.^1,2^ Concerns about inadequate access to blood during surgeries or lack of clear guidelines for blood requests often lead to excessive blood orders and subsequently, inappropriate distribution of blood products. In response to this issue, a program called the Maximum Blood Demand Model for Maximum Surgical Blood Order Scheduling (MSBOS) was introduced in the 1970s and 1980s to enhance the utilization of blood products.^3,4^

 By definition, MSBOS is a table of elective surgical procedures that lists the number of routinely pre-operatively cross-matched units of red blood cells and the number that are transfused for each procedure. This system simplifies blood ordering practices by providing a standard order for most patients and reducing the cross-match/transfusion ratio.^5^

 The concept of the cross-match/transfusion ratio was first introduced in 1975 by Henry Boral, who suggested that the ratio of cross-matched units to transfused units would be effective in the management of blood products.^6^ The American Association of Blood Banks recommends that this ratio should be equal to or lower than 2 for surgical patients and approximately 1 for medical patients.^7^

 In most medical centers, compatibility tests are thoroughly conducted before blood transfusions. This process makes the blood product unavailable to other patients for the next 48 hours after cross-matching, ultimately leading to improper distribution, wastage of blood products, and poor availability for emergency patients, as well as increased costs and workload for blood banks.^8^ Consequently, due to the limitations in the supply of blood products, timely transfusion for patients is not always achievable. Therefore, the proper use of these vital resources presents a significant challenge.^9^

 Given the importance of blood and its products, as well as the reported blood wastage in various studies, this study aimed to investigate the patterns of blood request and consumption at 5-Azar Educational Hospital, in the Golestan province.

## Materials and Methods

 The present study was a cross-sectional and descriptive investigation conducted at 5-Azar Educational Hospital, the trauma center of the Golestan province. It is important to note that the hospital is located directly across from the Iranian Blood Transfusion center in Gorgan city.

 For this purpose, we evaluated the number of blood products requested across various departments, the number of products transfused and not transfused, the ratio of cross-matched to transfused units (C/T), and the probability of blood transfusion index (T%) in each department of this general hospital. We also extracted the hemoglobin values prior to the first and second transfusions to gain insight into the physicians’ criteria for blood transfusion, categorizing the data according to department.

 Blood utilization indices were derived from the following equations^4^:

1. Cross-match transfusion ratio (C/T ratio) = Total number of units cross matched/total number of units transfused. Values of 2.5 and below indicate appropriate blood usage.
2. Transfusion probability (T%) = Total number of patients transfused /total number of patients cross matched × 100. Values of 30% and above indicate appropriate blood usage.

Due to high rate of blood request, the study population included all patients who had a blood reservation request during the period from June to August 2022. As a rule, all requests are recorded in the laboratory software as well as in the blood bank’s records, using the hospital admission number.

 The inclusion criteria comprised patients with a blood reservation request, while the exclusion criteria were incomplete patient records, specifically cases where the outcome of the blood reservation request was unavailable.

 After obtaining the necessary research approvals from the Research Council of Golestan University of Medical Sciences, we visited the blood bank department located in the laboratory of 5-Azar Educational Hospital in Gorgan with a referral letter. Due to the use of data recorded in the hospital software system, only the patients’ administration codes and blood transfusion information were documented, without including any patient names. Information on patients who had blood product reservations and transfusions was extracted through a checklist from their records.

 Statistical analysis was performed using SPSS version 20. Data are expressed as means ± standard deviation (SD). Comparisons between groups were conducted using chi-square (χ^2^) and ANOVA tests, and a *P *value of < 0.05 was considered statistically significant.

## Results

 In the present study, 514 patients with requests for blood products were included, comprising 281 men (54.7%) and 233 women (45.3%). The average age of the patients was 47.16 ± 20.42 years (range: 1-93 years) (Table 1). Second-time requests for blood products were registered for 225 patients. The mean hemoglobin level for patients’ first request was 9.27 ± 1.99 g/dL, while for their second request, it was 8.96 ± 1.54 g/dL, indicating no difference between them (*P* value = 0.215).

**Table 1:** Request of Blood Products and Follow-up for First and Second Requests by Patients

**Type of Product**		**First Transfusion**	**Second Transfusion**	***P*** ** Value**
Requested product	PC	420 (80.77%)	160 (68.67%)	0.021
	FFP	41 (7.88%)	28 (12.02%)	0.101
	Plt	57 (10.96%)	45 (19.31%)	0.078
	Cryo	2 (0.39%)	-	-

PC: Packed cell, FFP: Fresh frozen plasma, Plt: Platelet, Cryo: Cryoprecipitate.

 The distribution of patients receiving blood products by department indicated that the majority were hospitalized in the emergency departments (20.43%), followed by hematology and oncology (19.07%), and the intensive care unit (ICU) (18.09%). Furthermore, the frequency of blood product requests according to the specialist physician revealed that orthopedists (20.82%), oncologists (18.68%), and plastic surgeons (9.73%) had the highest number of requests for blood product reservations.

Table 2 shows the number of requests for blood products by department. As expected, packed red blood cells were the most commonly requested blood product. The highest number of requests for fresh frozen plasma (FFP) occurred in the burn department, followed by the ICU. The highest demand for platelets was in the hematology-oncology department, also followed by the ICU. Cryoprecipitate was requested only by the emergency department in two cases. The table also provides the number of requested units of each product across different departments for the second request; Packed cell (PC) requests decreased significantly in the second request in comparison to first request (*P* value = 0.021). Further details are presented in Table 2.

**Table 2:** Number of Requests for Blood Products by Requesting Departments

**Departments**			**First Transfusion **			**Second Transfusion **			
	**PC**	**FFP**	**Plt**	**Cryo**	**Total**	**PC**	**FFP**	**Plt**	**Total**
Surgery	53 (100%)	-	-	-	53	15 (100%)	-	-	15
Emergency	91 (86.7%)	4 (3.8%)	8 (7.6%)	2 (1.9%)	105	21 (77.8%)	2(7.4%)	4 (14.8%)	27
Oncology	65 (65.7%)	2 (2%)	32 (32.3%)	-	99	19 (36.5%)	2(3.8%)	31 (59.6%)	52
ICU	71 (74.8%)	12 (12.6%)	12 (12.6%)	-	95	34 (64.1%)	12(22.6%)	7 (13.2%)	53
Orthopedic	58 (98.3%)	-	1(1.7%)	-	59	25 (96.1%)	-	1 (3.9%)	26
Infectious	12 (92.3%)	1 (7.7%)	-	-	13	5 (100%)	-	-	5
Respiratory isolation	9 (81.8%)	1 (9.1%)	1(9.1%)	-	11	5 (83.3%)	-	1 (16.7%)	6
Neurosurgery	16 (88.9%)	1 (5.55%)	1(5.55%)	-	18	2 (100%)	-	-	2
Burn ICU	14 (51.8%)	11 (40.8%)	2(7.4%)	-	27	-	14 (70%)	6 (30%)	20
Burn	16 (64%)	9 (36%)	-	-	25	-	16 (72.7%)	6 (27.3%)	22
Other	15 (100%)	-	-	-	15	-	4 (80%)	1 (20%)	5

PC: Packed cell, FFP: Fresh frozen plasma, Plt: Platelet, Cryo: Cryoprecipitate.

**Figure 1:**
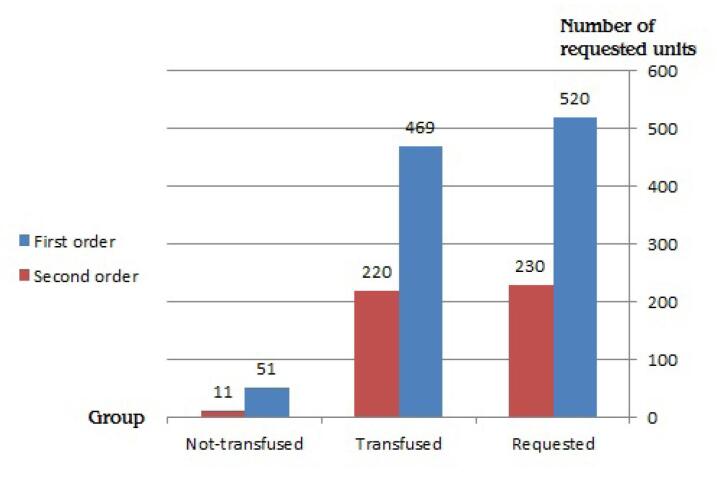
Comparison of Transfused and Non-transfused Blood Products in the First and Second Requests

 Evaluation of the non-transfused requested blood products showed that during the first request for blood products, a total of 520 requests for various types of blood products were recorded. In 51 cases, the requested products were not transfused. During the second request, a total of 231 requests for various types of blood products were recorded, of which 11 cases did not result in transfusion (Figure 1, Table 3).

**Table 3:** Status of Transfused and Non-Transfused Blood Products in the First and Second Requests

**Status**		**PC**	**FFP**	**Plt**	**Cryo**
First time	Transfusion	370 (88.1%)	41(100%)	56 (98.2%)	2 (100%)
	Not-transfused	50 (11.9%)	-	1 (1.8%)	-
Second time	Transfusion	153(95%)	32 (97%)	34 (94.4)	1(100%)
	Not-transfused	8 (5%)	1(3%)	2(5.6%)	-

PC: Packed cell, FFP: Fresh frozen plasma, Plt: Platelet, Cryo: Cryoprecipitate.

 Our findings also revealed that during the first request for blood products, the highest rate of non-transfused requested blood products was reported in the neurosurgery department (44.4%), followed by the surgery department (26.4%). In contrast, the burn department reported the lowest rate, with zero cases of non-transfused requested blood products.

 In the second request for blood products, the most cases of non-transfused requested blood products were observed in the respiratory isolation unit (20%), followed by the surgery department (13.3%) (Table 4).

**Table 4:** Status of Transfused and Non-Transfused Blood Products by Department

**Departments**	**First Time Request**		**Second Time Request**	
	**Transfused**	**Not-transfused**	**Transfused**	**Not-transfused**
Surgery	39 (73.6%)	14 (26.4%)	13 (86.7%)	2 (13.3%)
Emergency	94 (89.5%)	11 (10.5%)	25 (92.6%)	2 (7.4%)
Oncology	98 (99%)	1 (1%)	48 (96%)	2 (4%)
ICU	87 (91.6%)	8 (8.4%)	51 (94.4%)	3 (5.6%)
Orthopedic	55 (93.2%)	4 (6.8%)	25 (96.2%)	1 (3.8%)
Infectious	12 (92.3%)	1 (7.7%)	5 (100%)	-
Respiratory isolation	10 (90.9%)	1 (9.1%)	4 (80%)	1 (20%)
Neurosurgery	10 (55.6%)	8 (44.4%)	3 (100%)	-
Burn ICU	25 (92.6%)	2 (7.4%)	21 (100%)	-
Burn	25 (100%)	—	20 (100%)	-
Other	14 (93.3%)	1 (6.7%)	5 (100%)	-

**Figure 2:**
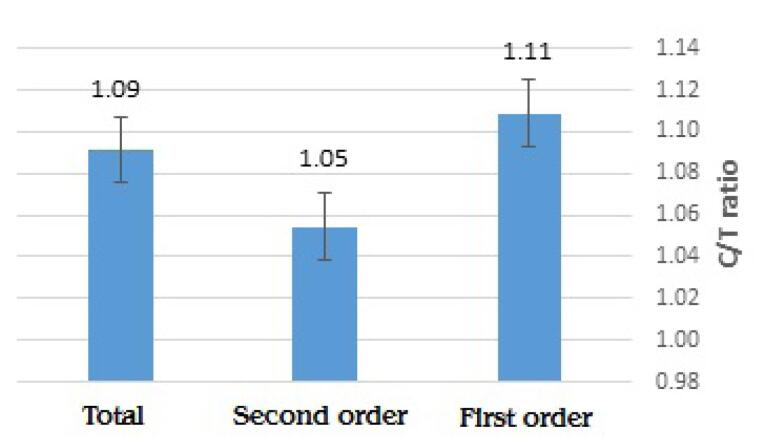
Ratio of Cross-Match to Transfusion (C/T) for the First Request, Second Request, and Total Requests

 The ratio of C/T for the first request, second request, and total requests is shown in Figure 2. There was no difference in C/T ratio between the first and second requests (*P* value = 0.525). As indicated, this ratio was 1.09 for the total number of blood product requests.

 The C/T ratio for the first request, second request, and total requests across different departments is also shown in Table 5. The highest C/T ratios were reported in the neurosurgery (1.67) and surgery (1.31) departments.

**Table 5:** Cross-Match to Transfusion (C/T) Ratio by Department

**Departments**	**C/T Ratio**		
	**First Time Request**	**Second Time Request**	**Total**
Surgery	1.36	1.15	1.26
Emergency	1.12	1.08	1.10
Oncology	1.01	1.04	1.03
ICU	1.09	1.06	1.08
Orthopedic	1.07	1.04	1.06
Infectious	1.08	1	1.04
Respiratory isolation	1.1	1.2	1.15
Neurosurgery	1.8	1	1.40
Burn ICU	1.08	1	1.04
Burn	1	1.05	1.03
Other	1.07	1	1.04
Total	1.16	1.05	1.11

**Figure 3:**
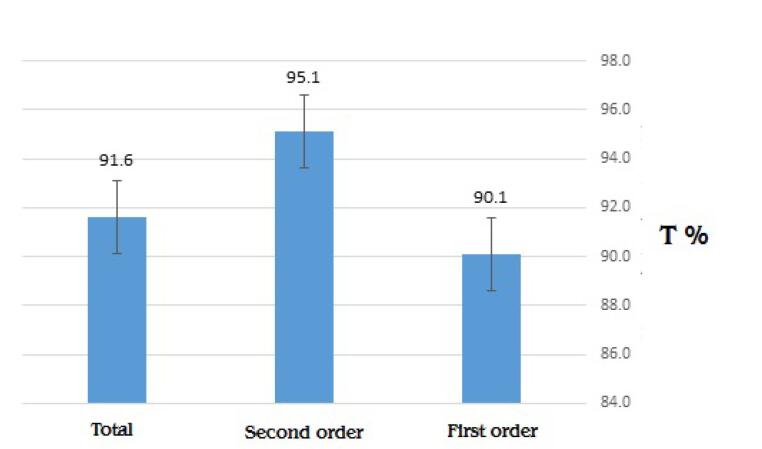
Transfusion Probability Index (T %) for the First Request, Second Request, and Total Requests

 The transfusion probability index (T %) for the first request, second request, and total requests is shown in Figure 3, indicating no difference between the first and second requests (*P* value = 0.201). The T % for the total number of blood product requests was 91.6%. The lowest probability of transfusion was associated with the neurosurgery department (57.9%), while the highest probability of transfusion was found in the burn department (100%) (Table 6).

**Table 6:** Transfusion probability index (T %) according to departments

**Departments**	**T%**		
	**First Request**	**Second Request**	**Total**
Surgery	73.6	86.7	76.5
Emergency	89.5	92.6	90.2
Oncology	99	95.9	98
ICU	91.4	94.2	92.4
Orthopedic	93.2	96.2	94.1
Infectious	92.3	100	94.4
Respiratory isolation	90.9	80	87.5
Neurosurgery	52.9	100	57.9
Burn ICU	92.3	100	95.7
Burn	100	100	100
other	93.3	100	95
Total	90.1	95.1	91.6

## Discussion

 The present study was conducted to determine the status of requests for and transfusions of blood products at 5-Azar educational hospital, a trauma center in the Golestan Province, in 2022. This hospital is strategically located directly across from the Iranian Blood Transfusion Center, allowing for rapid access to blood products with minimal complications.

 One of the primary challenges in conducting this study was the limited access to patient data, which had been archived due to hospital policies. Consequently, we were only able to monitor two months of the blood bank’s activities, focusing specifically on the receipt of requests, recording, and preparation of blood units for various departments. However, due to the multiple referrals of patients to this general and central hospital throughout the year, we managed to evaluate a total of 514 patients.

 The findings indicated that the Emergency, Hematology-Oncology, and ICU departments had the highest number of requests for blood product transfusions. Notably, orthopedic and oncology specialists accounted for the majority of these transfusions. For 43.8% of patients, a second order of blood products was requested. The C/T ratio, a key indicator of blood transfusion status, was found to be 1.09, with the highest ratios reported in the Neurosurgery and General Surgery departments.

 Many studies worldwide have examined similar indices related to blood consumption and transfusion practices. For instance, Tolyat and Barakchi^10^ reported that 20.2% of patients received blood transfusions, with a C/T ratio of 4.2, primarily in the Neurosurgery department, which aligns with our findings. In contrast, Nikpoor et al^11^ reported a C/T ratio of 1.33, with the highest transfusion rates occurring in the Cardiology department. Their study indicated an average of 2.43 units per patient, while our study reported this index as 1.5, suggesting a more appropriate level of blood product consumption in our setting compared to theirs.

 Patient blood management is defined as “the appropriate use of blood and blood components, with a goal of minimizing their use”.^12^ It represents an individualized, multidisciplinary approach to managing a patient’s blood, aimed at optimizing blood levels, minimizing blood loss, and enhancing tolerance to anemia following transfusion.^13^

 When comparing reports in the field of blood requests and consumption, contradictions in results are evident. These discrepancies may stem from the varying degrees of compliance with blood transfusion protocols within different healthcare systems, as well as differences across cities within our own country. In various studies, similar variations have been reported in blood usage patterns, emphasizing the need for standardized practices across institutions.^14-16^ Additionally, a systematic review highlighted the importance of protocol adherence in optimizing blood transfusion practices.^17^

 A primary concern in this area is understanding why blood product requisition and consumption need to be optimized despite existing guidelines. The challenge may lie in the gap between established guidelines and their practical implementation. Despite the availability of protocols for blood product requisition and consumption, there remains a pressing need for ongoing optimization and adherence to these standards. The contributing factors may include lack of awareness or training among healthcare providers,^18^ variations in practice across departments, and institutional barriers that hinder the effective adoption of best practices.^19-21^ To improve blood management practices, hospitals should focus on enhancing training programs for healthcare providers, ensuring that all staff are aware of and understand the guidelines for blood product requisition. Regular audits and feedback mechanisms could also be implemented to monitor compliance and identify areas for improvement. Furthermore, fostering a culture of collaboration between departments may facilitate better communication and adherence to transfusion protocols, ultimately leading to more efficient and appropriate use of blood products.

## Conclusion

 In conclusion, while our study provides valuable insights into blood product requests and transfusions at 5-Azar Educational Hospital, it also highlights the need for continued efforts to optimize blood management practices. By addressing the identified challenges and promoting adherence to guidelines, healthcare institutions can improve patient outcomes and ensure responsible use of blood resources.

## References

[1] Klein HG, Spahn DR, Carson JL (2007). Red blood cell transfusion in clinical practice. Lancet.

[2] Karbasian F, Zafari A, Goudarzipour K, Mesdaghi M, Shariatzadeh S, Eshghi P (2019). Evaluation of Changes in Blood Products Transfusion Indices Following Three Years of Implementation of Iranian Blood Transfusion Organization Standards. Iran J Blood Cancer.

[3] Hashemi J, Barzegar S, Soltani H, Nabavi A, Safdari M (2021). Evaluation of blood consumption pattern in Bojnurd Imam Ali hospital in 2020, North Khorasan province. J Clin Basic Res.

[4] Vibhute M, Kamath SK, Shetty A (2000). Blood utilisation in elective general surgery cases: requirements, ordering and transfusion practices. J Postgrad Med.

[5] Guidelines for implementation of a maximum surgical blood order schedule. The British Committee for Standards in Haematology Blood Transfusion Task Force. Clin Lab Haematol. 1990;12(3):321-7. 2272160

[6] Waheed S, Borhany M, Abid M, Naseer I, Shamsi T (2022). Blood ordering and transfusion practices: an insight toward better utility of blood products. Cureus.

[7] Basnet RB, Lamichhane D, Sharma VK (2009). A study of blood requisition and transfusion practice in surgery at Bir Hospital. Post-Graduate Medical Journal of NAMS.

[8] Peivandi Yazdi A, Alipour M, Jahanbakhsh SS, Gharavifard M, Taghavi Gilani M (2016). A survey of blood request versus blood utilization at a university hospital in Iran. Arch Bone Jt Surg.

[9] Sheikhansari S, Darbandi B, Zahiri Sorouri Z, Bagheralimi A (2015). Evaluating blood requests and transfusion practice in major surgical procedures. Iran J Blood Cancer.

[10] Tolyat M, Barakchi AA. Evaluation of blood utilization in Birjand Imam Reza hospital. Sci J Iran Blood Transfus Organ 2014;10(4):400-5. [Persian].

[11] Nikpoor AR, Daneshvar H, Sanei Moghaddam E, Askari M. Assessment of requisition and consumption indices of blood in educational hospitals in Kerman city. Sci J Iran Blood Transfus Organ 2013;10(1):12-9. [Persian].

[12] Tibi P, McClure RS, Huang J, Baker RA, Fitzgerald D, Mazer CD (2021). STS/SCA/AmSECT/SABM update to the clinical practice guidelines on patient blood management. J Extra Corpor Technol.

[13] Yazer MH, Triulzi DJ (2014). Things aren’t always as they seem: what the randomized trials of red blood cell transfusion tell us about adverse outcomes. Transfusion.

[14] Ambroise MM, Ravichandran K, Ramdas A, Sekhar G (2015). A study of blood utilization in a tertiary care hospital in South India. J Nat Sci Biol Med.

[15] Vimal M, Rakesh B, Anandabaskar N (2019). Pattern of utilization of blood and blood products in a tertiary care hospital. Recent Advances in Pathology and Laboratory Medicine.

[16] Alkhaldy HY, Alshahrani BS, Alkhaldi AY, Alqahtani AS, Muhayya I, Alqahtani M (2021). Patterns of blood products utilization at a tertiary care center in the southern region of Saudi Arabia. J Appl Hematol.

[17] Gilstad CW, Poisson J, Dubey R, Shariatmadar S, Jorgenson M, Gammon R (2023). The importance of patient blood management for patients, providers and the public. Ann Blood.

[18] Bolcato M, Russo M, Trentino K, Isbister J, Rodriguez D, Aprile A (2020). Patient blood management: the best approach to transfusion medicine risk management. Transfus Apher Sci.

[19] Mohammed AD, Ntambwe P, Crawford AM (2020). Barriers to effective transfusion practices in limited-resource settings: from infrastructure to cultural beliefs. World J Surg.

[20] Chegini A, Jamalian A, Abolhassani MR, Boroujerdi Alavi A (2024). A review of issues and challenges of implementation of patient blood management. Asian J Transfus Sci.

[21] Zaman B, Radmehr M, Sahraian A, Sohrabi P. Determination of the ratio and causes of unused blood ordered from blood bank blood in elective surgery in Rasoul-e-Akram hospital. Sci J Iran Blood Transfus Organ 2009;6(2):141-6. [Persian].

